# Sulbactam Protects Hippocampal Neurons Against Oxygen-Glucose Deprivation by Up-Regulating Astrocytic GLT-1 via p38 MAPK Signal Pathway

**DOI:** 10.3389/fnmol.2018.00281

**Published:** 2018-08-15

**Authors:** Jie Qi, Xiao-Hui Xian, Li Li, Min Zhang, Yu-Yan Hu, Jing-Ge Zhang, Wen-Bin Li

**Affiliations:** ^1^Department of Pathophysiology, Hebei Medical University, Shijiazhuang, China; ^2^Department of Science and Technology, Second Hospital of Hebei Medical University, Shijiazhuang, China; ^3^Neuroscience Center, Hebei Medical University, Shijiazhuang, China; ^4^Aging and Cognition Neuroscience Laboratory of Hebei Province, Shijiazhuang, China

**Keywords:** sulbactam, p38 MAPK, GLT-1, astrocyte, OGD, neuron-astrocyte co-culture, astrocyte culture

## Abstract

Sulbactam is an atypical β-lactam medication and reported to be neuroprotective by up-regulating glial glutamate transporter-1 (GLT-1) in rats. The present study was undertaken to study the role of p38 MAPK signal pathway in sulbactam induced up-regulation of GLT-1 expression in astrocytes and anti-ischemic effect. Neuron-astrocyte co-cultures and astrocyte cultures from neonatal Wistar rats were used. Cerebral ischemia was mimicked by oxygen-glucose deprivation (OGD). Hoechst (HO)/propidium iodide (PI) double fluorescence staining and 3-(4,5-dimethyl-2-thiazolyl)-2,5-diphenyl-2-H-tetrazolium bromide assay were used to evaluate neuronal death and cell viability, respectively. Immunocytochemistry and Western blot were used to detect protein expressions. Sulbactam pre-incubation significantly and dose-dependently prevented neuronal death and decline in cell viability induced by OGD in neuron-astrocyte co-cultures, and upregulated GLT-1 expression in astrocyte cultures endured OGD, which suggested that sulbactam might protect neurons against OGD by up-regulating astrocytic GLT-1 expression. It was further shown that the phosphorylated-p38 MAPK expression in astrocytes was up-regulated after the sulbactam pre-incubation and this up-regulation was moderate in amplitude. Especially, the time course of the up-regulation of phosphorylated-p38 MAPK was obviously earlier than that of GLT-1, which suggested possibility that p38 MAPK might be an upstream signal for GLT-1 up-regulation induced by sulbactam. We further found that SB203580, the specific inhibitor of p38 MAPK, dose-dependently inhibited the GLT-1 up-regulation induced by sulbactam either in non- or OGD-treated astrocytes and the protective effect of sulbactam on co-cultured neurons against OGD. Taken together, it might be concluded that sulbactam protects cerebral neurons against OGD by up-regulating astrocytic GLT-1 expression via p38 MAPK signal pathway.

## Introduction

The excessive release of glutamate after cerebral ischemia with insufficient clearance by glutamate transporters leads to accumulation of glutamate in synaptic cleft, which initiates excitotoxicity in brain (Fujikawa, 2015; Kritis et al., 2015). Glial glutamate transporter-1 (GLT-1) accounts for the most part in removing the extracellular glutamate (Kanai and Hediger, 1992; Pines et al., 1992; Fairman et al., 1995). A variety of evidence showed that up-regulation of GLT-1 expression and its uptake activity for glutamate could play neuronal protection against ischemic insult (Rothstein et al., 2005; Chu et al., 2007; Lipski et al., 2007; Harvey et al., 2011; Hu et al., 2015; Zhang et al., 2017). Especially, Rothstein et al. (2005) reported that ceftriaxone, a β-lactam antibiotic specifically upregulated GLT-1 expression and its uptake activity and showed clear neuronal protection against excitotoxicity of glutamate in oxygen-glucose deprivation (OGD) or amyotrophic lateral sclerosis models. These reports suggested a possibility using β-lactam medication for the prevention and treatment of brain ischemic diseases. However, some problems such as dysbacteriosis and bacterial resistance resulted from the powerful anti-bacterial effect of ceftriaxone limit its application in the prevention and treatment of brain ischemia in clinical practice. Sulbactam is an atypical β-lactam medication with little anti-bacterial capacity, which is usually used in combination with β-lactam antibiotics to potentiate their anti-bacterial effect. Our recent study has shown that sulbactam prevented ischemic injury of pyramidal neurons in the CA1 hippocampus by up-regulating GLT-1 in rat global cerebral ischemic model (Cui et al., 2015). These findings provided a beneficial basis and potential for clinical utilization of sulbactam in the prevention and treatment of cerebral ischemic injury because of the little anti-bacterial capacity and side effect of sulbactam. Therefore, it is important to elucidate signaling pathways involved in sulbactam-induced GLT-1 up-regulation and anti-ischemic effect for promoting the clinical application of sulbactam as an anti-cerebral ischemia medication.

As is well known, p38 mitogen-activated protein kinase (p38 MAPK) is an important intracellular signal transduction system and participates in a series of physiological and pathological processes including cell death or survival (Chang and Karin, 2001). Although some reports showed that activation of p38 MAPK might facilitate neuronal death after brain ischemic insult (Ozawa et al., 1999), beneficial or protective effects of moderate p38 MAPK activation has been well demonstrated in ischemic models of brain (Lennmyr et al., 2003; Blanquet et al., 2009; Cheng et al., 2016; Su et al., 2016) or other organs (Ruisong et al., 2015; Zhao et al., 2016), especially in ischemic preconditioning models (Park et al., 2001; Nishimura et al., 2003; Sun et al., 2010; Li et al., 2015). Therefore, the present study was undertaken to study the role of p38 MAPK signal pathway in the sulbactam-induced up-regulation of GLT-1 expression in astrocytes during the process of anti-ischemic effect of sulbactam.

## Materials and Methods

### Animals

The present study was performed using neuron-astrocyte co-cultures and astrocyte cultures from postnatal 0–1-day Wistar rats provided by the Experimental Animal Center of Hebei Medical University. The pregnant and neonatal rats were housed with standard chow and water *ad libitum* in ambient temperature of 22 ± 2°C and kept under a 12 h/12 h light/dark cycle with the light on at 07:00 a.m. All animal care and experimental procedures were performed in accordance with approved guidelines of the National Institutes of Health for the Care and Use of Laboratory Animals, and the guidelines approved by the Committee of Ethics on Animal Experiments of Hebei Medical University. All efforts were made to minimize suffering and the number of animals used in the study.

### Experimental Design and Groupings

#### Part 1. The Effect of Sulbactam on Neuronal Survival and GLT-1 Expression in Astrocytes After OGD

Steady primary neuron-astrocyte co-cultures for 10 days and astrocyte cultures at three or four generations were randomly divided into the following four groups (*n* = 5, which means five independent cultures, the same in the following in each group and subgroup).

**Figure 1:**
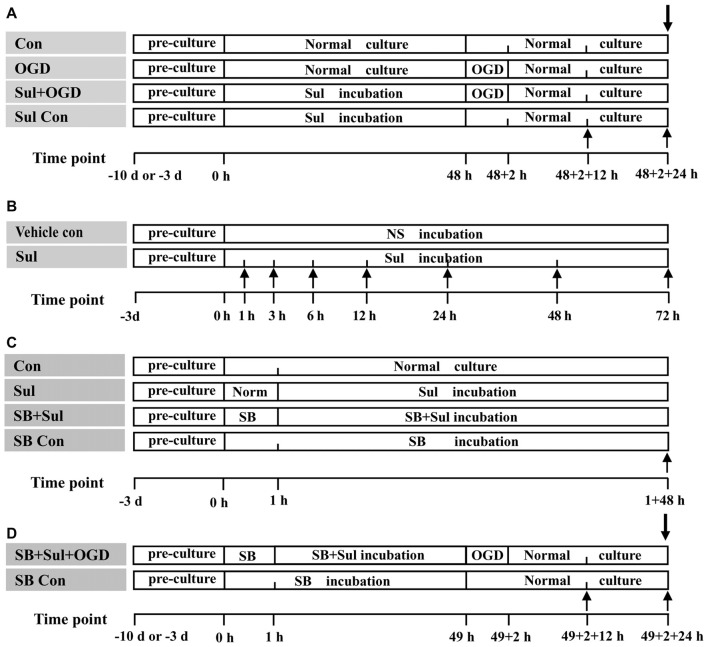
The schematic illustration of experimental protocols in each group. Abbreviations: Con, control; OGD, oxygen-glucose deprivation; NS, normal saline; Sul, sulbactam; SB, SB203580, HO/PI, hoechst/propidium iodide; MTT, 3-(4,5-dimethylthiazol-2-yl)-2,5-diphenyltetrazolium bromide. **(A)** is for Part 1. **(B)** is for Part 2. **(C)** is for Part 3. **(D)** is for Part 4. The upward arrows indicate the time points when the GLT-1 and p38 MAPK expressions in the astrocyte cultures were assayed with immunocytochemistry and western blot analysis. The downward arrows indicate the time point when the neuronal survival and viability in neuron-astrocyte co-cultures were assayed with HO/PI staining and MTT method.

1. **Control group**: the neuron-astrocyte co-cultures and astrocyte cultures were maintained in normal medium for 48 h + 2 h + 24 h (Figure 1A), which were corresponded to the times for sulbactam incubation, OGD and recovery after re-oxygenation from OGD, respectively, in the following groups.
2. **OGD group**: first, the neuron-astrocyte co-cultures and astrocyte cultures were kept under normal medium for 48 h. After that, the cultures were endured OGD for 2 h and then conducted a recovery cultured for 24 h under normal condition (Figure 1A).
3. **Sulbactam+OGD group**: first, the neuron-astrocyte co-cultures and astrocyte cultures were maintained for 48 h under the presence of sulbactam (dissolved in normal saline (NS)) in the final concentrations of 5 μM, 25 μM and 125 μM in the cultures. Then the cultures were endured OGD free of sulbactam for 2 h. Whereafter, a recovery culture was continued with fresh normal medium free of sulbactam for 24 h under normal condition (Figure 1A). In addition, a NS+OGD group was designed as the sulbactam’s vehicle control group, in which only NS was administrated instead of sulbactam.
4. **Sulbactam control group**: this group was designed only as control for neuronal survival and viability in the neuron-astrocyte co-cultures. The co-cultures were maintained under 125 μM sulbactam for 48 h and then kept in the fresh normal medium free of sulbactam for 2 + 24 h (Figure 1A).

The neuronal death including necrosis and apoptosis in the neuron-astrocyte co-cultures was evaluated by Hoechst (HO)/propidium iodide (PI) staining, and the neuronal survival was evaluated with 3-(4,5-dimethylthiazol-2-yl)-2, 5-diphenyltetrazolium bromide (MTT) method at 24 h after re-oxygenation from OGD. The GLT-1 expression in the astrocyte cultures was assayed by immunocytochemistry and western blot analysis at 12 h and 24 h after re-oxygenation from OGD (Figure 1A).

#### Part 2. The Comparison Between the Time Course of GLT-1 and Phosphorylated p38 MAPK Expressions After Sulbactam Incubation in Normal Treated Astrocytes

The astrocyte cultures were used in this part. First, to determine the effect of sulbactam on GLT-1, phosphorylated p38 MAPK and total p38 MAPK, dose dependency of these protein expressions to sulbactam was observed. Sulbactam in a final concentration of 5 μM, 25 μM and 125 μM respectively was added to the cultures (*n* = 5 in each dose). The astrocytes were harvested at 3 h for phosphorylated-p38 MAPK, at 24 h for total p38 MAPK and at 48 h for GLT-1 expression after the sulbactam incubation. The reason for selection of these time points is due to the relative high expression of these proteins after sulbactam incubation in our preliminary experiments. Vehicle control groups were designed, in which only NS was administrated instead of sulbactam.

Then, to determine the time course of the effect of sulbactam on these proteins, the astrocytic cultures were incubated in medium with 125 μM sulbactam and harvested at 1 h, 3 h, 6 h, 12 h, 24 h, 48 h and 72 h after the sulbactam incubation (Figure 1B; *n* = 5 in each time point). Vehicle control groups were designed, in which only NS was administrated instead of sulbactam.

The expressions of GLT-1, phosphorylated and total p38 MAPK were assayed by immunocytochemistry and western blot analysis.

#### Part 3. The Effect of p38 MAPK Inhibition on Sulbactam-Induced GLT-1 Expression in Non- and OGD-Treated Astrocytes

##### The GLT-1 Expression in Non-OGD Treated Astrocytes

This part was performed using astrocyte cultures and consisted of the following four groups (*n* = 5 in each group and subgroup):

1. **Control group**: astrocyte cultures were maintained in normal medium for 1 h + 48 h (Figure 1C), which were corresponded to the time for SB203580 and sulbactam incubations respectively in the following groups.
2. **Sulbactam group**: astrocyte cultures were maintained in normal medium for 1 h and then were incubated in medium with 125 μM sulbactam for 48 h (Figure 1C).
3. **SB203580+sulbactam group**: first, astrocyte cultures were administrated with SB203580 (dissolved in DMSO) alone for 1 h, and then administrated with sulbactam in final concentration of 125 μM, and co-incubated with SB203580 for 48 h (Figure 1C). According to the incubation concentration of SB203580, this group was further divided into 2.5 μM, 5 μM and 10 μM subgroups. In addition, a DMSO+sulbactam group was designed as the SB203580’s vehicle control group.
4. **SB203580 control group**: the cultures were administered with SB203580 in a concentration of 10 μM and maintained for 1 h + 48 h (Figure 1C).

After the above treatments, the GLT-1 expression in the astrocytes was evaluated with immunocytochemistry and western blot analysis.

##### The GLT-1 Expression in OGD-Treated Astrocytes

This part was performed using astrocyte cultures as well and consisted of the control, OGD, sulbactam+OGD, and SB203580+sulbactam+OGD groups (*n* = 5 in each group and subgroup). The protocols for control, OGD, sulbactam+OGD groups were the same as those in the corresponding groups in Part 1 (Figure 1A), except only one concentration of sulbactam in 125 μM was added. The protocols for SB203580+sulbactam+OGD group consisted of the following procedures (Figure 1D): First, the protocol for the administration of SB203580+sulbactam was the same as that in SB203580+sulbactam group in part 3.1. Then, the subsequent OGD and oxygen recovery was the same as that in OGD group. According to the incubation concentration of SB203580, this group was further divided into 2.5 μM, 5 μM and 10 μM subgroups. In addition, a DMSO+sulbactam+OGD group was designed as the SB203580’s vehicle control group.

After the above treatments, GLT-1 expression in the astrocytes at 12 h and 24 h after the re-oxygenation from OGD was evaluated with immunocytochemistry and western blot analysis.

#### Part 4. The Effect of p38 MAPK Inhibition on the Neuronal Protective Effect of Sulbactam Against OGD in Neuron-Astrocyte Co-cultures

This part was performed using neuron-astrocyte co-cultures and consisted of control, OGD, sulbactam+OGD, SB203580+sulbactam+OGD and SB203580 control groups (*n* = 5 in each group). The protocols of control, OGD, sulbactam+OGD, SB203580+sulbactam+OGD were the same as those in the corresponding group in the part 3.2. The cultures in SB203580 control group were administered with SB203580 in a concentration of 10 μM and maintained for 1 h + 48 h followed by a subsequent normal culture for 2 + 24 h (Figure 1D). Cells were harvested at 24 h after the re-oxygenation from OGD or corresponding time point in the control and SB203580 control groups (Figure 1D). The neuronal survival condition was evaluated with HO/PI and MTT methods.

### Neuron-Astrocyte Co-culture

Neuron-astrocyte co-culture was prepared according to the method described previously (Kaech and Banker, 2006). Briefly, under anesthesia with isoflurane, neonatal Wistar rats were decapitated. The bilateral hippocampi were isolated and digested with 2 mg ml^−1^ papain plus 0.1 mg ml^−1^ DNase for 30 min at 37°C. The single cells isolated mechanically were adjusted to 1 × 10^6^ ml^−1^ and plated onto 12-well plates. After culture for 6 h–8 h in Dulbecco’s modified Eagle’s medium (DMEM) containing 10% fetal bovine serum, the medium was replaced with Neurobasal-A (Gibco, Gaithersburg, MD, USA) plus 2% B27 (Gibco, Gaithersburg, MD, USA) and 2 mM L-glutamine without any antibiotics. The oxygen concentration in the incubator was normoxia (20%), and high-glucose (25 mM) was maintained in the medium for neuronal survival and growth (Kaech and Banker, 2006). Half of the medium was replaced with fresh medium once every 2 to 3 days. Steady primary neuron-astrocyte co-cultures for 10 days were used for the study. This experimental protocol for the co-culture succeeded in co-growing of neurons and astrocytes. The proportion of neurons to astrocytes was about 1–2:1 as counted according to cell morphology and distribution (Supplementary Figures S1, S2). This proportion of neurons to astrocytes in the co-cultures was similar to previous reports, in which neurons were protected against OGD by the up-regulation of GLT-1 in neuron- astrocyte co-cultures (Hurtado et al., 2002; Romera et al., 2004).

### Astrocyte Culture

The method to gain single cells of the hippocampus was the same as that of neuron-astrocyte co-cultures. The single cells were adjusted to 2 × 10^5^ ml^−1^ and plated onto 25 cm^2^ flask. Astrocyte cultures were maintained in 10% fetal bovine serum DMEM and replaced with the whole fresh medium once every 3 days. Astrocytes were purified by oscillation at 260 rpm for 18 h and cell passage cultivation to remove oligodendroglia, microglia and neurons. The third or fourth generation of the purified astrocyte cultures was used for experiments. The purity of the astrocyte cultures was >98% as determined by glial fibrillary acidic protein immunocytochemistry analysis.

### OGD

The primary hippocampal neuron-astrocyte cultures and astrocyte cultures were exposed to OGD according to the method described previously (Fernandes et al., 2014). The culture was conducted with glucose-free DMEM and humidified atmosphere with 94% N_2_/1% O_2_/5% CO_2_ for 2 h at 37°C in tri-gas incubator (3131, ThermoFisher Scientific, Waltham, MA, USA). After the OGD for 2 h, the medium was replaced with normal medium for neuron-astrocyte cultures or astrocyte cultures in a normoxic conditions.

### Neuronal Death Assay

Neuronal death was assessed by double-staining with PI (membrane-impermeable) and HO 33258 (membrane-permeable). Briefly, after incubation with PI (1 μg ml^−1^) for 15 min, cells were washed and fixed with 4% paraformaldehyde. After permeabilization with 0.2% Triton X-100, the cells were incubated with HO (0.1 μg ml^−1^) solution for 5 min. The red or blue fluorescence were captured under inverted fluorescent microscopy (DP 72, Olympus, Japan) with exciting light at 488 nm and 350 nm, respectively. The red (PI positive), or small bright blue (intensive HO positive) fluorescent images indicate dead cells. Live cells are weak HO positive only and stained dark blue with the normal shape of cell nucleus. Experiments were repeated at least five times for each different cell culture preparation, and three wells were plated for each group in each time. The average percentage of dead cells was calculated. Counting visual fields were selected randomly by a blind observer. Three different visual fields per well were evaluated, and 50–100 total cells at a minimum were counted in each field.

In general, 2 h OGD could not cause the death of astrocytes (Goldberg and Choi, 1993; Sochocka et al., 1994; Reichert et al., 2001). Our preliminary experiments also showed similar results that the dead cells after 2 h OGD showed the neuronal characteristics in morphology (Supplementary Figures S1, S2). So, the percent of dead cell counted above in the present study actually reflects the death of neurons in the co-cultures.

### Cell Viability Assay

MTT can detect the activity of succinate dehydrogenase in mitochondria and then to be used to measure cell viability. The assay was conducted as previously described (Aldasoro et al., 2016). The co-cultures of neuron-astrocytes in 24-well plate were incubated with MTT (0.5 mg ml^−1^ in final concentration; Sigma-Aldrich China Inc., China) for 4 h at 37°C. MTT-formazan in cells was solubilized by DMSO and was measured at 570 nm by spectrophotometer (Sunrise TW, UK). Cell viability was represented by the ratio of the optical density in treatment groups vs. that in control group. Cell cultures were repeated at least five times for each group, and each measurement consisted of six wells for each different cell culture preparation.

For the same reasons mentioned above in Neuronal Death Assay, the decrease of cell viability actually reflected the injury and death of neurons in the co-culture.

### Western Blot Analysis

The astrocytes were homogenized in lysis buffer including phosphate kinase inhibitors and other proteinase inhibitors. The protein concentration of the supernatant collected was determined by bicinchoninic acid method. Proteins (50 μg for each sample) with loading buffer were electrophoresed on a 12% SDS-PAGE gel and transferred to polyvinylidene difluoride membranes (Millipore Corporation, Billerica, MA, USA) by semi-dry transfer. GLT-1 is a glycosylated protein that usually migrates bands in 71 kd and 110 kd in electrophoresis. Based on the previous report (Sha et al., 2017) and our preliminary experiment, we chose the 71 kd of GLT-1 as the target of observation. After covering unspecific antigens by immersing the membranes in 5% skim milk, the membranes were incubated respectively with the primary antibodies overnight at 4°C (GLT-1: 1:2,000, guinea-pig derived polyclonal antibody, Lot 2015978, Millipore, Billerica, MA, USA; phosphorylated-p38 MAPK: 1:2,000, mouse derived polyclonal antibody, Lot 612280, BD Pharmigen, San Jose, CA, USA; p38 MAPK: 1:4,000, rabbit derived polyclonal antibody, Lot GTX110720, Gene Tex, Irvine, CA, USA; β-actin: 1:4,000, mouse derived monoclonal antibody, Lot GTX629630, Gene Tex, Irvine, CA, USA). The membranes were then incubated with biotin-labeled secondary antibody for 1 h at 37°C (GLT-1: 1:5,000, biotinylated anti-guinea-pig IgG, Lot AP108B, Chemicon International Inc., Temecula, CA, USA; phosphorylated-p38 MAPK and β-actin: 1:4,000, biotinylated anti-mouse IgG, Lot 16-18-06, KPL, USA; p38 MAPK: 1:4,000, biotinylated anti-rabbit IgG, Lot 16-18-06, KPL, Carlsbad, CA, USA), and horseradish peroxidase-conjugated streptavidin (1:4,000, Lot 434323, Invitrogen, USA) for 1 h at 37°C. The labeled bands were developed using ECL, visualized with Odyssey Fc Imager (Odyssey GS-710, Gene Company Limited, USA), and analyzed with an image analyzer (Alpha Image, 2200, Alpha, USA).

### Immunocytochemistry Analysis

For p38 MAPK and phosphorylated-p38 MAPK immunocytochemistry staining, cells were permeabilized in 0.3% Triton X-100, while for GLT-1 staining, this procedure was omitted because GLT-1 is a kind of membrane protein. Endogenous peroxidases were blocked by 3% H_2_O_2_ solution. The primary antibodies were the same as those used in western blot analysis except the concentration was 1:2,000 for GLT-1, 1:500 for phosphorylated-p38 MAPK and 1:500 for p38 MAPK. After being rinsed, cell slides were incubated with horseradish peroxidase-conjugated IgG secondary antibody working solution (GLT-1 and p-p38 MAPK: Lot PV-9000 ZSGB-BIO, China; p38 MAPK: Lot PV-9001 ZSGB-BIO, China). Peroxidase activity was developed using a DAB substrate kit (Lot 12196A11, ZSGB-BIO, China).To ensure consistency of reagent incubation time and developing time, the slides from all groups were stained in the same immunocytochemistry run.

### Data Analysis

The experimental data were processed with statistical software Statistical Package for the Social Science (SPSS) 16.0 manufactured by IBM Corporation. Data were presented as mean ± SD and were tested using one-way ANOVA between the different groups. The Fisher’s least significant difference (LSD) was used for pairwise comparison among groups. A probability of *P* < 0.05 was considered significant for all statistical analyses. The data and statistical analysis comply with the recommendations on experimental design and analysis in pharmacology (Curtis et al., 2015).

## Results

### Sulbactam Protects Neurons and Up-Regulates Astrocytic GLT-1 Expression Against OGD

#### Neuronal Survival and Viability

**Figure 2:**
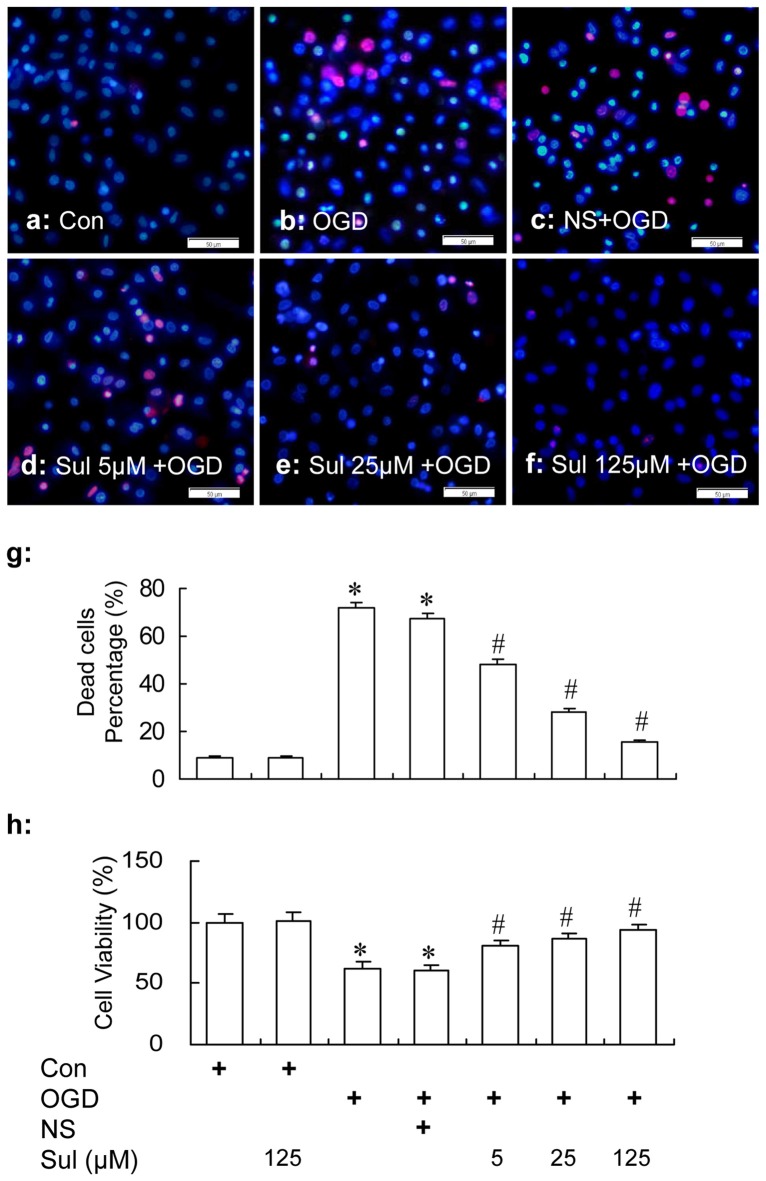
HO/PI staining **(a–g)** and MTT **(h)** assays show the protection of sulbactam incubation against OGD in primary hippocampal neuron-astrocyte co-cultures. Abbreviations: Con, control; OGD, oxygen glucose deprivation; NS, normal saline; Sul, sulbactam; HO/PI, hoechst/propidium iodide; MTT, 3-(4,5-dimethylthiazol -2-yl)-2, 5-diphenyltetrazolium bromide. The scale bar in the each representative photomicrographs of HO/PI staining **(a–f)** is 50 μm. *P < 0.05 vs. control group, ^#^*P* < 0.05 vs. OGD or vehicle group. The observation was performed at 24 h after re-oxygenation from OGD. It is shown that sulbactam incubation effectively prevents the neuronal death and decrease in cell viability induced by OGD in a dose dependent manner.

HO/PI staining showed that sulbactam reduced neuronal death induced by OGD in a dose dependent manner in hippocampal neuron-astrocyte co-cultures. In Figure 2, it could be found that there were a lot of living neurons stained in dark blue with normal shape of cellular nucleus in control group (Figure 2a). OGD induced many neuronal deaths which were stained in red (PI positive) or bright blue (intensive HO positive) with small nuclei because of pyknosis (Figure 2b), and the percent of neuronal death was significantly increased compared with the control group (Figure 2g). Sulbactam incubation effectively prevented the neuronal death induced by OGD in a dose dependent manner (Figures 2d–g). The administration of sulbactam at 125 μM had the best drug efficacy (Figures 2f,g). The percentage of neuronal death in Sulbactam (125 μM)+OGD group reduced about 70% compared with the OGD group (Figures 2f,g). Administration of vehicle instead of sulbactam had no effect on the OGD-induced neuronal death (Figures 2c,g). In addition, sulbactam in the sulbactam control group had no effect on the percentage of neuronal death (Figure 2g, photomicrograph is not shown) compared with control group. MTT assay showed that sulbactam incubation in the sulbactam+OGD group dose-dependently prevented the decrease in cell viability induced by OGD in neuron-astrocyte co-cultures (Figure 2h). Sulbactam incubation in the sulbactam control group had no effect on the cell viability of normal co-cultures compared with control group (Figure 2h).

#### GLT-1 Expression

**Figure 3:**
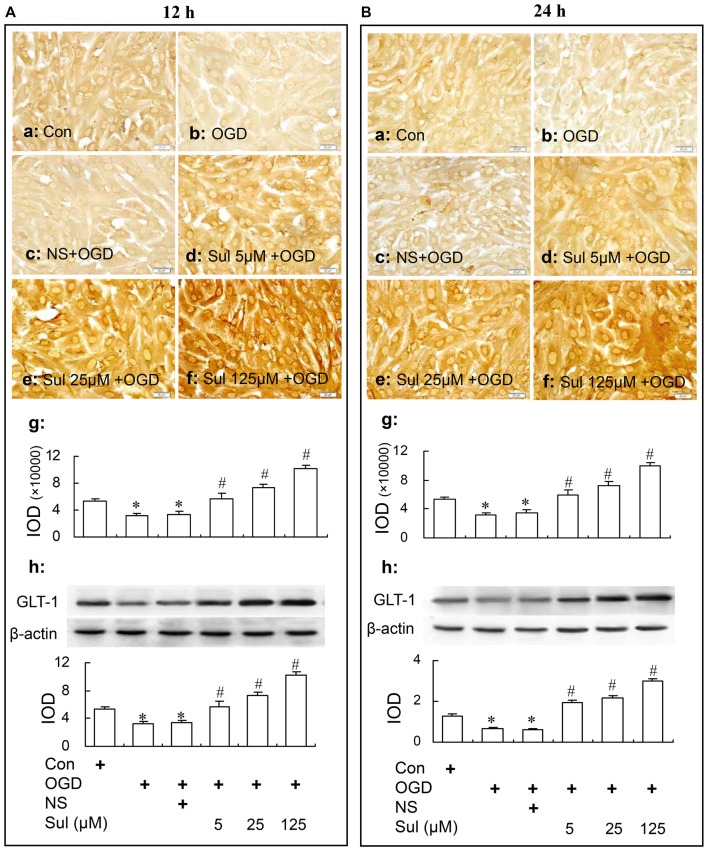
Immunocytochemistry and western blot analysis show the up-regulating effect of sulbactam incubation on GLT-1 expression in OGD-treated astrocytes at 12 h **(A)** and 24 h **(B)** after re-oxygenation from OGD in astrocyte cultures. Abbreviations: Con, control; OGD, oxygen glucose deprivation; NS, normal saline; Sul, sulbactam; IOD, integral optical density. The insets of **(a–f)** are representative photomicrographs of immunocytochemical staining in each group and the scale bar on them is 20 μm. The inset of **(g)** is the quantitative presentation of the immunostaining density with IOD. The inset of **(h)** is the results of western blot analysis. The upper portion shows the immunoblot bands and the lower portion is the quantitative presentation of the immunoblots with the ratio of IOD of the immunoblotting bands of GLT-1 to β-actin. *P < 0.05 vs. control group, ^#^*P* < 0.05 vs. OGD group or vehicle group. It is shown that sulbactam incubation for 48 h significantly up-regulates the astrocytic GLT-1 expression in a dose dependent manner at either 12 h or 24 h after re-oxygenation from OGD.

Immunochemical assay and western blot showed that sulbactam up-regulated astrocytic GLT-1 expression against OGD in a dose dependent manner in astrocyte cultures. As shown in Figure 3, there was a basic expression shown as brown GLT-1 immunoparticles in astrocyte culture in control group (Figures 3Aa,Ba) in immunocytochemistry assay. Compared with control group, the GLT-1 expression was downregulated in OGD group (Figures 3Ab,Ag,Bb,Bg) and sulbactam incubation significantly up-regulated the GLT-1 expression in a dose dependent manner at both 12 h (Figures 3Ad–f,Ag) and 24 h (Figures 3Bd–f,Bg) after OGD in sulbactam+OGD group. In the large concentrations of 25 μM and 125 μM, the sulbactam incubation made the expression exceeded the control level about 1.5–2 folds (Figures 3Ae–g,Be–g). The vehicle for sulbactam had no effect on GLT-1 protein expression levels in OGD-treated astrocytes (Figures 3Ac,g,Bc,g). Western blot analysis showed similar results in the expression of GLT-1 after the sulbactam incubation in the OGD-treated astrocytes at the time point of either 12 h (Figure 3Ah) or 24 h (Figure 3Bh).

### Sulbactam Up-Regulates Phosphorylated-p38 MAPK Expression Earlier Than GLT-1 Expression in Normal Astrocytes

#### GLT-1 Expression

**Figure 4:**
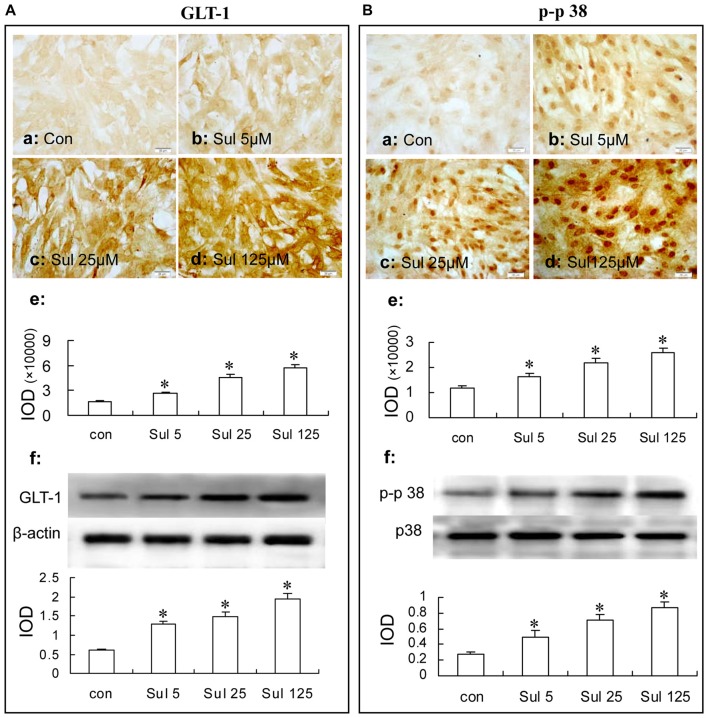
Immunocytochemistry and western blot analysis show the dose dependance of sulbactam on astrocytic GLT-1 **(A)** and phosphorylated-p38 MAPK **(B)** up-regulation in normal astrocyte cultures. Abbreviations: Con, control; Sul, sulbactam; p-p38, phosphorylated-p38 MAPK; p38, total p38 MAPK; IOD, integral optical density. The insets of **(a–d)** are representative photomicrographs of immunocytochemical staining in each group and the scale bar on them is 20 μm. The inset of **(e)** is the quantitative presentation of the immunostaining density with IOD. The inset of **(f)** is the results of western blot analysis. The upper portion shows the immunoblot bands and the lower portion is the quantitative presentation of the immunoblots with the ratio of IOD of the immunoblotting bands of GLT-1 to β-actin or p-p38 to total p38. *P < 0.05 vs. control group. It is shown that sulbactam incubation significantly up-regulates GLT-1 and p-p38 expressions in a dose dependent manner.

In Figure 4A, immunocytochemistry assay showed that after sulbactam incubation of 5 μM, 25 μM and 125 μM for 48 h, the GLT-1 immunoparticles significantly increased and showed a dose-dependency (Figures 4Aa–e). Western blot analysis showed a similar dose-dependency in the up-regulation of GLT-1 expression after the sulbactam incubation (Figure 4Af).

**Figure 5:**
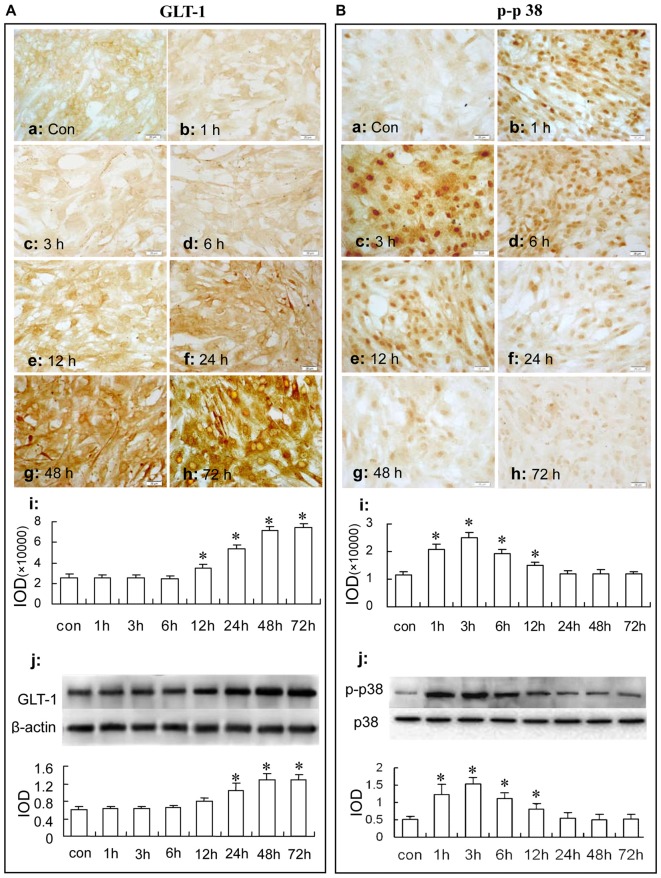
Immunocytochemistry and western blot analysis show the time course of astrocytic GLT-1 **(A)** and p-p38 up-regulation **(B)** under sulbactam incubation (125 μM) in normal astrocyte cultures. Abbreviations: Con, control; p-p38, phosphorylated-p38 MAPK; p38, total p38 MAPK; IOD, integral optical density. The insets of **(a–h)** are representative photomicrographs of immunocytochemical staining in each group and the scale bar on them is 20 μm. The inset of **(i)** is the quantitative presentation of the immunostaining density with integrated optical density. The inset of **(j)** is the results of western blot analysis. The upper portion shows the immunoblot bands and the lower portion is the quantitative presentation of the immunoblots with the ratio of IOD of the immunoblotting bands of GLT-1 to β-actin or p-p38 to total p38. *P < 0.05 vs. control group. It is shown that sulbactam up-regulates phosphorylated-p38 MAPK earlier than GLT-1 expression in normal astrocytes.

Then, we observed the time course of the up-regulation of GLT-1 expression after 125 μM sulbactam incubation. In Figure 5A, it was found with immunocytochemistry assay (Figures 5Aa–i) and western blot analysis (Figure 5Aj) that the GLT-1 up-regulation started at 12 h or 24 h, reached the peak at 48 h and maintained the high level till 72 h after sulbactam incubation.

#### p38 MAPK Expression

**Figure 6:**
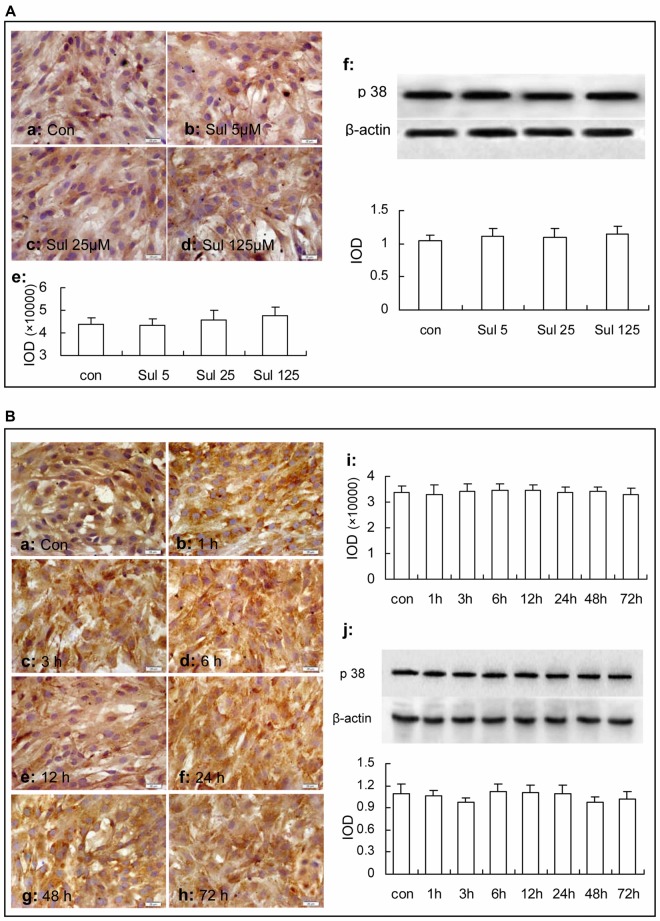
Immunocytochemistry and western blot analysis show effects of sulbactam incubation on astrocytic total p38 MAPK expression in normal astrocytes. Abbreviations: Con, control; Sul, sulbactam; p38, total p38 MAPK; IOD, integral optical density. The insets of **(a–d)** in **(A)** and **(a–h)** in **(B)** are representative photomicrographs of immunocytochemical staining in each group and the scale bar on them is 20 μm. The inset of **(e)** in **(A)** and the inset of **(i)** in **(B)** are the quantitative presentations of the immunostaining density with IOD. The inset of **(f)** in **(A)** and the inset of **(j)** in **(B)** are the results of western blot analysis. The upper portion shows the immunoblot bands and the lower portion is the quantitative presentation of the immunoblots with the ratio of IOD of the immunoblotting bands of total p38 MAPK to β-actin. It is shown that no changes in total p38 MAPK expression are found under sulbactam incubation in any concentration or any time point observed.

In Figure 4B, immunocytochemistry assay showed that phosphorylated-p38 MAPK expression was mainly located in the nucleus of the cultured astrocytes (Figures 4Ba–d). There were a few phosphorylated-p38 MAPK immunoparticles in the vehicle control group (Figure 4Ba) in the cultured astrocytes. Compared with the vehicle control group, the expression of phosphorylated-p38 MAPK was up-regulated after the 5 μM, 25 μM and 125 μM sulbactam incubation for 3 h and showed a clear dose-dependency (Figures 4Bb–d,e). Western blot analysis showed a similar effect and dose-dependency in the up-regulation of phosphorylated-p38 MAPK expression after the sulbactam incubation (Figure 4Bf). Either immunocytochemistry or western blot assay did not show changes in the expression of p38 MAPK after sulbactam incubation for 24 h in any one of the concentrations of 5 μM, 25 μM and 125 μM (Figures 6Aa–f).

Then, we observed the time course of the up-regulation of phosphorylated-p38 MAPK and p38 MAPK after sulbactam incubation with immunocytochemistry assay (Figures 5Ba–i) and western blot analysis (Figure 5Bj). In Figure 5B, it was shown that the up-regulation of phosphorylated-p38 MAPK expression started at 1 h, reached the peak at 3 h and fallen to the baseline at 24 h after the sulbactam incubation. However, the expression of p38 MAPK did not show significant changes after the 125 μM sulbactam incubation at any time point observed by immunocytochemistry and western blot assay (Figures 6Ba–j).

### The Inhibition of p38 MAPK Activation Reduced the GLT-1 Up-Regulation Induced by Sulbactam in Both Non- and OGD-Treated Astrocytes

#### In Non-OGD Treated Astrocytes

**Figure 7:**
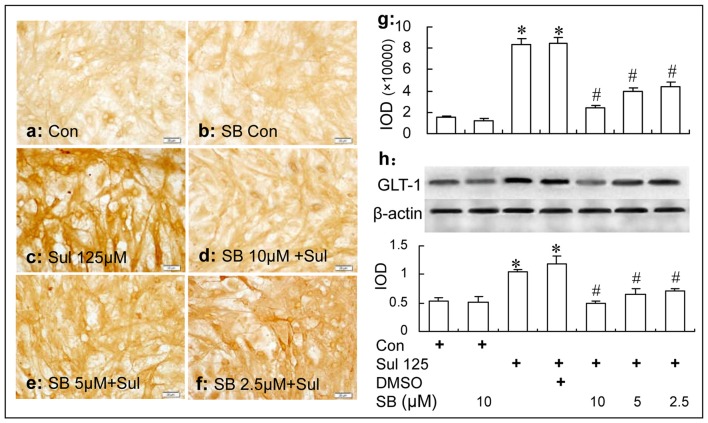
Immunocytochemistry and western blot analysis show the effect of p38 MAPK inhibition by SB203580 on astrocytic GLT-1 up-regulation induced by sulbactam (125 μM for 48 h) in non-OGD treated astrocyte cultures. Abbreviations: Con, control; Sul, sulbactam; DMSO, dimethyl sulphoxide; SB, SB203580. IOD, integral optical density. The insets of **(a–f)** are representative photomicrographs of immunocytochemical staining in each group and the scale bar on them is 20 μm. The inset of **(g)** is the quantitative presentation of the immunostaining density with IOD. The inset of **(h)** is the results of western blot analysis. The upper portion shows the immunoblot bands and the lower portion is the quantitative presentation of the immunoblots with the ratio of IOD of the immunoblotting bands of GLT-1 to β-actin. *P < 0.05 vs. vehicle control group, ^#^*P* < 0.05 vs. sulbactam group. It is shown that p38 MAPK inhibition by SB203580 dose-dependently inhibits the astrocytic GLT-1 up-regulation induced by the sulbactam incubation in non-OGD treated astrocytes.

Immunocytochemistry and western blot analysis showed that the GLT-1 up-regulation induced by sulbactam incubation was dose dependently inhibited by the specific p38 MAPK inhibitor, SB 203580 in the astrocyte culture. It was also shown in Figure 7 that compared with control group, GLT-1 expression was up-regulated by sulbactam incubation with the concentration of 125 μM in sulbactam group (Figures 7c,g,h). Compared with sulbactam group, administration of 2.5 μM, 5 μM and 10 μM SB203580 inhibited GLT-1 up-regulation induced by sulbactam (Figures 7d–h) in a dose dependent manner. Vehicle (DMSO) for SB 203580 had no inhibited effect on GLT-1 up-regulation by sulbactam (Figures 5g,h, photomicrograph of immunocytochemistry is not shown). The basic expression of GLT-1 was not significantly inhibited by the administration of SB 203580 in SB 203580 control group (Figures 7b,g,h).

#### In OGD-Treated Astrocytes

**Figure 8:**
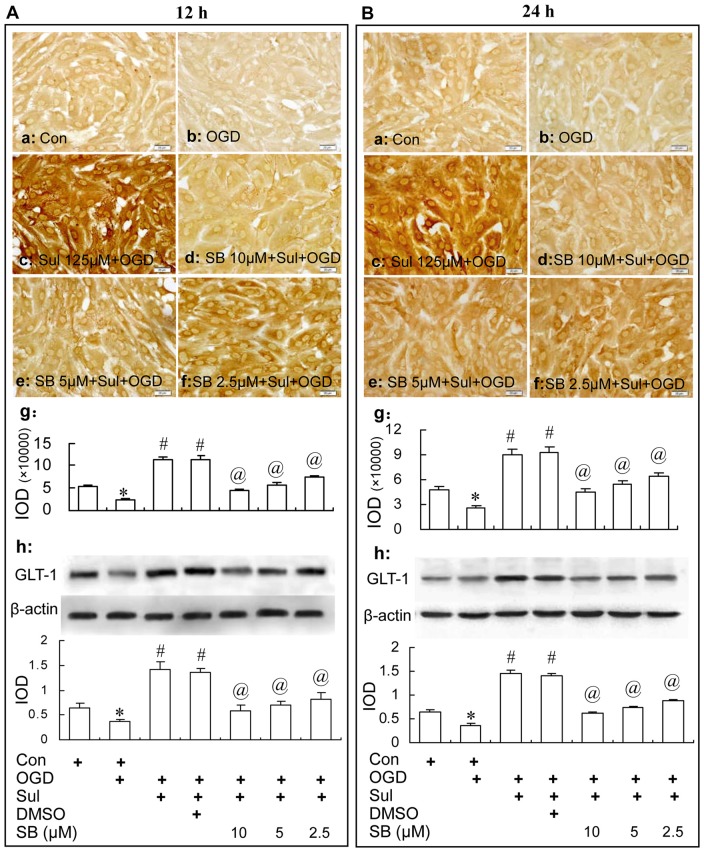
Immunocytochemistry and western blot analysis show the effect of p38 MAPK inhibition by SB203580 on GLT-1 up-regulation induced by sulbactam incubation (125 μM) in OGD-treated astrocytes at 12 h **(A)** and 24 h **(B)** after re-oxygenation from OGD in astrocyte cultures. Abbreviations: Con, control; OGD, oxygen glucose deprivation; Sul, sulbactam; DMSO, dimethyl sulphoxide; SB, SB203580, IOD, integral optical density. The insets of **(a–f)** are representative photomicrographs of immunocytochemical staining in each group and the scale bar on them is 20 μm. The inset of **(g)** is the quantitative presentation of the immunostaining density with IOD. The inset of **(h)** is the results of western blot analysis. The upper portion sows the immunoblot bands and the lower portion is the quantitative presentation of the immunoblots with the ratio of IOD of the immunoblotting bands of GLT-1 to β-actin. *P < 0.05 vs. control group; ^#^*P* < 0.05 vs. OGD group; ^@^*P* < 0.05 vs. sulbactam+OGD group. It is shown that the p38 MAPK inhibition by SB203580 dose-dependently inhibits the astrocytic GLT-1 up-regulation induced by the sulbactam incubation in OGD-treated astrocytes.

Immunocytochemistry and western blot analysis showed that the GLT-1 up-regulation induced by sulbactam in OGD-treated astrocytes was dose dependently inhibited by SB 203580 as well (Figure 8). Specifically, compared with sulbactam+OGD group at 12 h (Figure 8Ac,g,h) and 24 h (Figures 8Bc,g,h), the administration of SB 203580 in the group of SB203580+sulbactam+OGD inhibited the GLT-1 up-regulation induced by sulbactam in astrocytes either at 12 h (Figures 8Ad–h) or 24 h (Figures 8Bd–h) after OGD in a dose dependent manner. Vehicle (DMSO) for SB 203580 had no inhibited effect on the GLT-1 up-regulation induced by sulbactam in the OGD-treated astrocytes as well (Figures 8Ag–h, 8Ag–h, Bg–h, photomicrographs of immunocytochemistry are not shown).

### The Inhibition of p38 MAPK Activation Eliminated the Neuronal Protection of Sulbactam Against OGD in Neuron-Astrocyte Co-cultures

**Figure 9:**
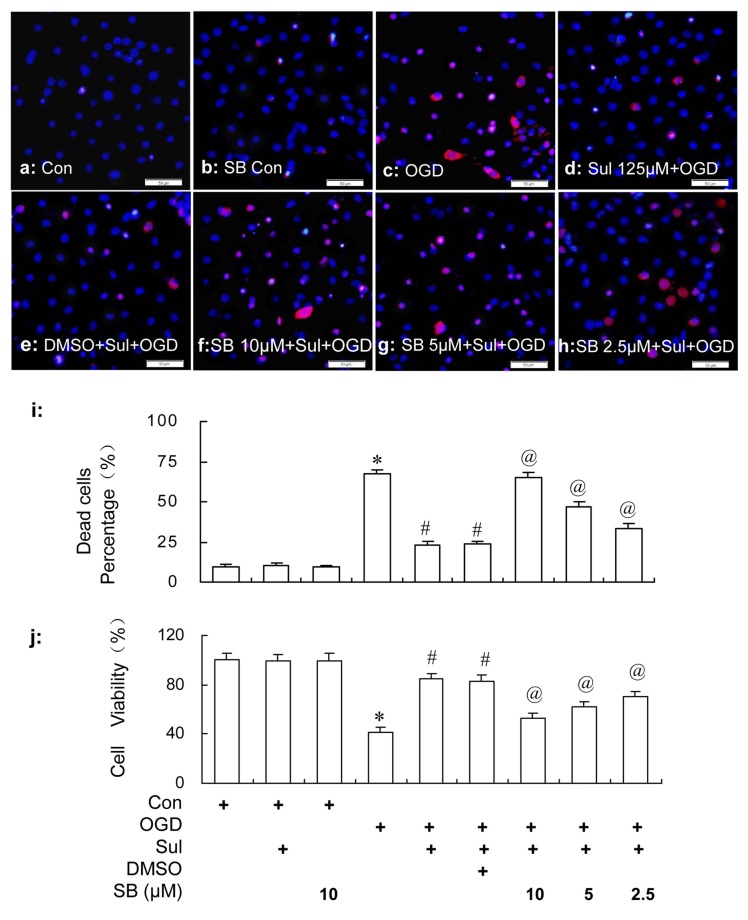
HO/PI staining **(a–i)** and MTT **(j)** assays show the effect of p38 MAPK inhibition by SB203580 on the neuronal protective effect of sulbactam (125 μM) against OGD in primary hippocampal neuron-astrocyte co-cultures. Abbreviations: Con, control; OGD, oxygen glucose deprivation; Sul, sulbactam; DMSO, dimethyl sulphoxide; SB, SB203580; HO/PI, hoechst/propidium iodide; MTT, 3-(4,5-dimethylthiazol-2-yl)-2,5-diphenyltetrazolium bromide. The scale bar in the each representative photomicrographs of HO/PI staining **(a–h)** is 50 μm. *P < 0.05 vs. control group; ^#^*P* < 0.05 vs. OGD group; ^@^*P* < 0.05 vs. sulbactam+OGD group. The observation was performed at 24 h after re-oxygenation from OGD in the neuron-astrocyte co-cultures. It is shown that SB203580 effectively inhibits the neuronal protective effect of sulbactam against OGD in a dose dependent manner, which represents with the increased neuronal death and decline in cell viability after the SB203580 treatment in SB+sulbactam+OGD group compared with sulbactam+OGD group.

Consistent with the results in the “Neuronal Survival and Viability” section, it was shown in Figure 9 by HO/PI staining that sulbactam incubation of 125 μM effectively prevented the neuronal death induced by OGD in the neuron-astrocyte co-cultures (Figures 9d,i). Compared with sulbactam+OGD group, 2.5 μM, 5 μM and 10 μM SB203580 significantly inhibited the neuronal protection of the sulbactam incubation against OGD in a dose dependent manner in the SB203580+sulbactam+OGD group (Figures 9f–h,i) The vehicle (DMSO) for SB203580 had no effect on the neuronal protection of sulbactam incubation against OGD (Figures 9e,i). In addition, 10 μM SB203580 alone in did not increase neuronal death (Figures 9b,i) in SB control group compared with control group. MTT assays (Figure 9j) showed that sulbactam incubation of 125 μM significantly prevented the decrease in the cell viability against OGD in the neuron-astrocyte co-cultures. Specifically, SB203580 inhibited the increase in cell viability by sulbactam in a dose dependent manner in SB203580+sulbactam+OGD group. The vehicle for SB203580 had no effect on the increase of cell viability induced by the sulbactam. 10 μM SB203580 alone in the SB203580 control group had no effect on the cell viability compared with control group.

## Discussion

The present study is a part of our series of studies about the anti-ischemic effect of sulbactam. Our recent study has shown in a rat global cerebral ischemic model that sulbactam could prevent cerebral ischemic injury by up-regulating GLT-1 (Cui et al., 2015). For better understanding the role of sulbactam and promoting its clinical application study as an anti-cerebral ischemia medication, the primary aim of the present study was to elucidate the role of p38 MAPK played in the sulbactam-induced neuronal protection and GLT-1 up-regulation against ischemia. It might be difficult *in vivo* study to discern whether the up-regulation of GLT-1 after sulbactam treatment was directly mediated by p38 MAPK activation in astrocytes because activity of astrocytes may be influenced by neurons in some extent (Verkhratsky, 2010; Barreto et al., 2011; Parpura et al., 2012). Therefore, the present study was performed using neuron-astrocyte co-cultures for neuronal protective experiments and using astrocyte cultures for experiments on GLT-1 expression.

In order to elucidate the hypothesis that sulbactam protects neurons by directly up-regulating astrocytic GLT-1, we first confirmed the neuronal protective effect of sulbactam against OGD using neuron-astrocyte co-cultures. We found that pre-incubation with sulbactam effectively prevented neuronal death induced by OGD in the neuron-astrocyte co-cultures. This result is similar to the protective effect of ceftriaxone (the typical β-lactam medication) reported by Rothstein et al. (2005), in which ceftriaxone (1 μM in concentration) incubation for 48 h reduced neuronal death induced by OGD in cortical co-cultures. Concerning the selection of sulbactam concentrations in this experiment, we comprehensively referenced Rothstein et al.’s (2005) report and the sulbactam’s clinical application. It is illustrated in drug instruction of sulbactam that the peak concentration of sulbactam is about 0.5–12 μg mL^−1^ (1.96–47 μM) in cerebrospinal fluid 1–4 h after 1 g sulbactam is intravenously infused to patients with bacterial meningitis (Foulds et al., 1983). According to these data, we selected three concentrations of 5 μM, 25 μM and 125 μM and observed that the sulbactam’s effect of anti-OGD was dose dependent. The results well illustrated the anti-OGD effect of sulbactam and supported mutually with our previous study *in vivo* (Cui et al., 2015). Although post-treatment with medication for stroke patients is generally expected, studies have indicated the importance of preconditioning and tolerance against cerebral ischemic injury (Dirnagl et al., 2009), particularly for some predictable ischemic events such as cardiopulmonary bypass surgery, carotid endarterectomy, or transient ischemic attack. However, it should be noticed that the magnitude of the protective effect by sulbactam post-treatment was much weaker than that by sulbactam pre-treatment protocols (Cui et al., 2015). The focus of our present study was on the mechanisms of the up-regulation of GLT-1 and the protective effect on neurons induced by sulbactam. Therefore, the protocol of sulbactam pre-treatment was chosen to make the protective effect and up-regulation more prominent and exact.

To investigate the role of astrocytic GLT-1 up-regulation in the anti-OGD effect of sulbactam, we observed the effect of sulbactam on astrocytic GLT-1 expression using astrocyte cultures. It was found that sulbactam pre-treatment not only prevented the downregulation of GLT-1 expression induced by OGD, but also up-regulated the expression to more than 1.5–2 fold compared to control group. It is noteworthy that the up-regulation by sulbactam persisted to 24 h after re-oxygenation from OGD, which covered the time when the neuronal injury took place after the OGD in neuron-astrocyte co-cultures. This time characteristic of astrocytic GLT-1 up-regulation is extraordinarily beneficial to play the protective effect on neurons against OGD. In our previous study *in vivo*, it was found that sulbactam up-regulated GLT-1 expression at the transcriptional and translational levels (Cui et al., 2015). However, the microenvironments within the brain and the cross talks among different types of cells were complex, and it was difficult to clarify whether sulbactam could directly stimulate the transcription and translation of GLT-1 in astrocytes. The astrocyte cultures in the present study could significantly simplify the microenvironment and directly show the effect of sulbactam on the astrocytic GLT-1 expression. Therefore, our above findings indicated the direct up-regulating effect of sulbactam on astrocytic GLT-1 and strongly suggested the role of the astrocytic GLT-1 up-regulation in the anti-OGD effect of sulbactam.

Like a double-edged sword, the activation of p38 MAPK signal pathway can mediate not only protection against injury but also pathophysical damages (Chang and Karin, 2001). In most cases, heavy and lasting activation of p38 MAPK to fierce and harmful stimulus such as ischemic insult, reactive oxygen species etc., might result in release of harmful factors and mediate injuries (McDonald et al., 1998; Piao et al., 2000; Sugino et al., 2000; Lu et al., 2011), while moderate and temporary activation of p38 MAPK to mild stress such as ischemic preconditioning might result in release of protective factors and mediate cellular or organic protection (Lennmyr et al., 2003; Nishimura et al., 2003; Tabakman et al., 2005; Sun et al., 2010; Zhao et al., 2013). For example, in our previous report it was shown that after lethal brain ischemic stimulation the phosphorylated-p38 MAPK up-regulated extremely, in which the peak level was more than four folds of the basal level and the up-regulation lasted a relative long period to 7 days. However, after cerebral or limb ischemic preconditioning, the phosphorylated-p38 MAPK expression only up-regulated moderately with the peak level just about two folds of the basal level peaked at 3 h and 6 h and the up-regulation recovered on 1–4 days after the stimulation (Sun et al., 2010; Zhang et al., 2017). It was also reported that cardiac preconditioning up-regulated phosphorylated-p38 MAPK about two folds of the basal level and reduced infarct size in wild type B6 mouse (Sicard et al., 2010). In the present study, the phosphorylated-p38 MAPK expression peaked at 3 h with about two folds of the basal level, and fell to basal level at 24 h after sulbactam incubation inastrocyte cultures. These characteristics resembled those induced by cerebral or limb ischemic preconditioning, which indicated that the activation of p38 MAPK in astrocytes induced by sulbactam was gentle, which would lead to advantageous signal transduction to the neuronal protection of sulbactam.

Thus, we further compared the time course between the GLT-1 and phosphorylated-p38 MAPK expression after sulbactam incubation in astrocyte cultures. The up-regulation of GLT-1 by sulbactam incubation started at 12 h and lasted at high level for 48 h and 72 h at least. This characteristic was similar to that in ceftriaxone-treated cultures in Rothstein et al.’s (2005) report. However, the up-regulation of phosphorylated-p38 MAPK induced by sulbactam incubation occurred significantly earlier than GLT-1 up-regulation in either the start or peak time point. Therefore, the present results provided the possibility that the activation of p38 MAPK might be the upstream in the up-regulation of GLT-1 by sulbactam in astrocytes.

On the basis of above observation, we then investigated the effect of inhibition of p38 MAPK on the sulbactam induced GLT-1 up-regulation and neuronal protection against OGD. SB203580, a highly specific, cell-permeable inhibitor of p38 MAPK, could bind competitively to the ATP binding site of p38 MAPK to inhibit the activation of downstream pathway of p38 MAPK (Lee et al., 1994; Cuenda et al., 1995; Bain et al., 2003). In our present study, SB203580 showed a significant dose-dependent inhibition on the up-regulation of GLT-1 under normal and OGD conditions in cultured astrocytes and neuroprotection in the co-culture of neuron-astrocytes. These results indicated that activation of p38 MAPK participated in the signal mediation of the sulbactam-induced GLT-1 up-regulation and neuronal protection against OGD. A large number of studies used SB203580 as inhibitor of the p38 MAPK to study the p38 MAPK mediated regulation on target proteins and neuronal apoptosis (Hwang et al., 2016; Livne-Bar et al., 2016). In addition, we designed a SB Control group, in which no cell death was observed. The small doses of SB we selected according to previous reports could not induce cell death (Nishimura et al., 2003; Zhao et al., 2013). These reports and results ensured that the non-specific effects and influence on targets especially in the context of cell death regulation were limited.

Taken together, it can be concluded that activation of p38 MAPK participated in the signal mediation of the sulbactam-induced GLT-1 up-regulation and neuronal protection against OGD. Further confirmation *in vivo* study that the inhibition of phosphorylated-p38 MAPK eliminates the sulbactam-induced GLT-1 up-regulation and neuronal protection would reinforce the above conclusion.

Activation of p38 MAPK pathway has been implicated in a variety of stress response such as inflammation, heat, and oxidative stress (Ono and Han, 2000; Kyriakis and Avruch, 2001; Johnson and Lapadat, 2002). For understanding the mechanism of sulbactam-induced anti-OGD, it is particular important to elucidate how is p38 MAPK activated by upstream pathway and how does the activation of p38 MAPK induce the up-regulation of GLT-1 after the treatment of sulbactam. p38 MAPKs are mainly activated by upstream kinases (MAPKK) by phosphorylation of the threonine-glycine-tyrosine residues motif, although the mechanism of autophosphorylated activation is also reported (Han et al., 1994; Ota et al., 2010). The classical pathway for p38 MAPK activation is mediated by the cascades of MAP3Ks and MAP2Ks as upstream kinases. The MAP3Ks include MAP kinase kinase kinases (MEKK1–4), mixed-lineage kinase 2/3 (MLK2/3), TGFβ-activated kinase (TAK1), thousand-and-one amino acid 1–3 (TAO1–3), and apoptosis signal-regulating kinase 1/2 (ASK1/2). The MAP2Ks include MKK 3/6 and 4 (Cuadrado and Nebreda, 2010). Non-classical activation of p38 MAPK via autophosphorylation including TAK1-binding protein 1 (TAB1), ZAP70 and HSP90/Cdc37 was also reported (Ge et al., 2002; Tanno et al., 2003; Salvador et al., 2005; Ota et al., 2010). These pathways might be involved in the activation of p38 MAPK after sulbactam treatment in the present study. Activated p38 is rapidly translocated to the nucleus, and activates many downstream kinases and transcription factors, such as cAMP response element-binding protein, NF-κB, etc., which were reported to contribute to the up-regulation of astrocytic GLT-1 transcription (Schulze-Osthoff et al., 1997; Gallo and Johnson, 2002; Ghosh et al., 2011; Lee et al., 2012; Parpura et al., 2012; Karki et al., 2013, 2014). Therefore, these signal transduction molecules might be involved in the elevated GLT-1 expression after activation of p38 MAPK by sulbactam in astrocytes in the present study. However, further systematic studies are necessary to prove the speculations.

Because sulbactam has little antibacterial capacity and side effects when used alone, we believe the present studies to be of particular relevance and significance for clinical practice in the prevention and treatment of cerebral ischemic injury using sulbactam as an anti-cerebral ischemic medication.

## Author Contributions

W-BL, MZ, Y-YH, JQ and X-HX conceived and designed the study. JQ, X-HX, LL and J-GZ performed the experiments. JQ and LL analyzed the data. JQ, Y-YH and W-BL contributed to drafting and critical revision of the manuscript. All authors read and approved the manuscript.

## Conflict of Interest Statement

The authors declare that the research was conducted in the absence of any commercial or financial relationships that could be construed as a potential conflict of interest.

## Abbreviations

DMSO: Dimethyl sulphoxide
GLT-1: Glial glutamate transpor-1
HO/PI: Hoechst/Propidium iodide
IOD: Integral optical density
MTT: 3-(4,5-Dimethylthiazol-2-yl)-2,5-Diphenyltetrazolium bromide
NS: Normal saline
OGD: Oxygen-glucose deprivation
p38 MAPK: p38 Mitogen activated protein kinase
p-p38 MAPK: Phosphorylated-p38 mitogen activated protein kinase.
