# Supplementary material for: Sulbactam Protects Hippocampal Neurons Against Oxygen-Glucose Deprivation by Up-Regulating Astrocytic GLT-1 via p38 MAPK Signal Pathway
Source: Front Mol Neurosci. 2018 Aug 15;11:281. doi: 10.3389/fnmol.2018.00281 (PMC6104165; doi:10.3389/fnmol.2018.00281)
Supplement: Supplementary file 1 [file Data_Sheet_1.pdf]

Supplementary Material

Sulbactam Protects Hippocampal Neurons against Oxygen-glucose Deprivation by Up-regulating Astrocytic GLT-1 via p38 MAPK Signal Pathway

Jie Qi[1], Xiao-Hui Xian[1#], Li Li[2], Min Zhang[1], Yu-Yan Hu[1], Jing-Ge Zhang[1], Wen-Bin Li[1, 3,*]

1. Department of Pathophysiology, Hebei Medical University, Shijiazhuang, P.R. China.

2. Department of Science and Technology, Second Hospital of Hebei Medical University, Shijiazhuang, P.R.China.

3. Aging and Cognition Neuroscience Laboratory of Hebei Province, Shijiazhuang, P.R. China.

#. Co-first author

* **Corresponding Author:** Department of Pathophysiology, Hebei Medical University, Shijiazhuang, P.R. China, and Aging and Cognition Neuroscience Laboratory of Hebei Province, Shijiazhuang, P.R. China. E-mail: liwbsjz@163.com

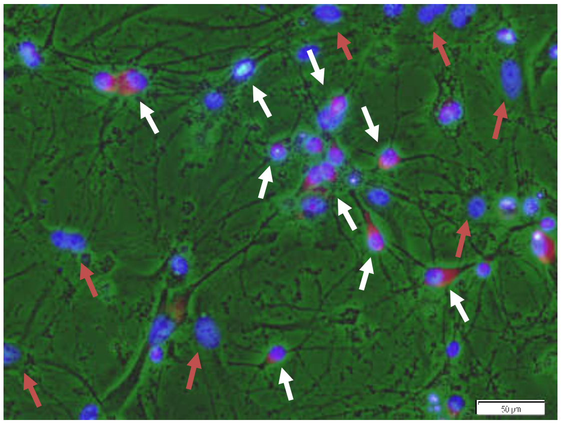

Supplementary Figure 1. The figure is merged by photographs of HO staining, PI staining and bright field in the same visual field in neuron-astrocyte co-culture at 24 h after 2 h OGD. Astrocytes (pointed by red arrows) have thick branching processes and big round nucleus, and tightly attach to the bottom of the dish like a carpet. The nuclei of astrocytes are shown moderate hoechst-positive stain (the dark blue-stain) with large, uniform granular sensation and clear nucleoli, which indicate the survival condition of the astrocytes. The neurons like scattered pearls float in the upper layer of astrocytes (pointed by white arrows), and show halo around and three-dimensional sense cell body, slender protrusions and small nucleus. The PI positive (red stain) and/or condensed hoechst positive stain (bright blue stain) show clear morphological characteristics of dead neurons. The proportion of neurons to astrocytes is about 1~2:1. The percent of astrocytes and the extensive and close contact between astrocytes and neurons are beneficial for the neuronal protection by astrocytes. The results also support the traditional view that 2 h OGD can only cause the death of neurons but not astrocytes.

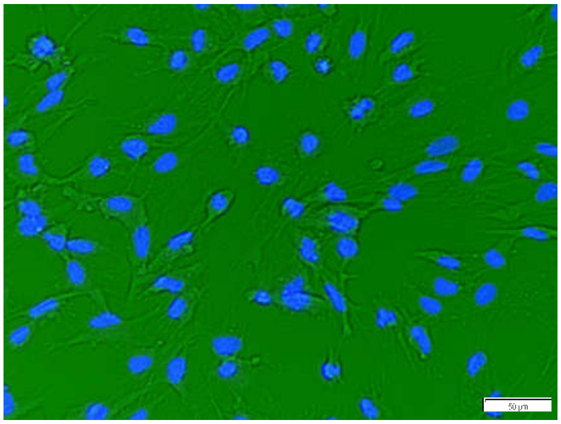

Supplementary Figure 2. The figure is merged by photographs of HO staining, PI staining and bright field in the same visual field in astrocyte culture at 24 h after 2 h OGD. The nuclei of the astrocytes are stained as moderate hoechst positive in dark blue with normal shape and granular sensation, which indicate the characteristics of survival astrocytes. This finding indicates that almost all of the astrocytes survive at 24 h after 2 h OGD.
